# An experimental study on providing a scientific evidence for seven-time alcohol-steaming of Rhei Rhizoma when clinically used

**DOI:** 10.1186/s12906-015-0922-y

**Published:** 2015-10-27

**Authors:** Yeomoon Sim, Hyein Oh, Dal-Seok Oh, Namkwon Kim, Pil Sung Gu, Jin Gyu Choi, Hyo Geun Kim, Tong Ho Kang, Myung Sook Oh

**Affiliations:** Department of Life and Nanopharmaceutical Science, Graduate School, Kyung Hee University, 26 Kyungheedae-ro, Dongdaemun-gu, Seoul 02447 Republic of Korea; Division for Medical Research, Korea Institute of Oriental Medicine, Daejeon, Republic of Korea; Department of Oriental Pharmaceutical Science, College of Pharmacy and Kyung Hee East–west Pharmaceutical Research Institute, Kyung Hee University, 26 Kyungheedae-ro, Dongdaemun-gu, Seoul 02447 Republic of Korea; Department of Oriental Medicinal Materials & Processing, College of Life Sciences, Kyung Hee University, Gyeonggi-do, Republic of Korea

**Keywords:** Rhei Rhizoma, Alcohol-steaming, Hepatotoxicity, Dongeuibogam

## Abstract

**Background:**

Rhei Rhizoma (RR) has been widely used as laxative and processed to alter its therapeutic actions or reduce its side effects. In this study, we evaluated experimentally the clinical application guideline that RR should be alcohol-steamed seven times before being used in elderly patients, as described in Dongeuibogam, the most famous book on Korean traditional medicine.

**Methods:**

Unprocessed RR (RR-U) was soaked in rice wine, steamed and then fully dried (RR-P1). The process was repeated four (RR-P4) or seven times (RR-P7). Reversed-phase high-performance liquid chromatography was used to determine the RR-U, RR-P1, RR-P4 and RR-P7 (RRs) constituents. To evaluate the effect of RRs on liver toxicity, human hepatoma cells (HepG2) were treated with RRs at 100 μg/mL for 4 h and then cell viabilities were measured using the 3-(4,5-dimethylthiazol-2-yl)-2,5-diphenyltetrazolium bromide method. To confirm the effects *in vivo*, 5-week-old male Sprague–Dawley rats were treated with RRs at 3 g/kg/day for 21 days. Body weight and serum biochemical parameters were measured and liver histology was assessed.

**Results:**

The levels of sennosides decreased in processed RRs in an iteration-dependent manner, while the emodin level was unaffected. In HepG2 cells, cell viability was reduced with RR-U, while the toxicity decreased according to the number of processing cycles. The changes in body weight, relative liver weight and liver enzymes of RR-U-treated rats were reduced in processed RRs-treated rats. Histopathological analysis indicated swelling and cholestasis improved following seven times alcohol-steaming cycles.

**Conclusions:**

These results provide experimental evidence that RR-P7 almost completely reduces RR hepatotoxicity.

**Electronic supplementary material:**

The online version of this article (doi:10.1186/s12906-015-0922-y) contains supplementary material, which is available to authorized users.

## Background

Rhei Rhizoma (RR) is a rhubarb rhizome and belongs to the genus *Rheum* in the family Polygonaceae. According to traditional literature, RR has a bitter taste and a cold property and has been commonly used to remove accumulation with purgation, clear heat and purge fire, cool the blood and remove toxins, expel stasis to unblock the meridian and drain dampness in Korea, China, Japan and other Asian countries [1]. RR contains dianthrone glycosides (sennosides A-F) and anthraquinones (chrysophanol, emodin, aloe-emodin, physcion and rhein) [2]. Several studies have investigated the pharmacological effects of RR, such as its purgative and cathartic, anti-inflammatory, anti-oxidative and anti-microbial effects [3]. Although RR has been widely used for its various pharmacological actions, it is reported that RR at high doses is toxic to the liver and kidney [4, 5].

The processing of medicinal herbs is a common pharmaceutical technique that allows the use of different therapeutic modalities based on traditional theory; the most important purpose of herb processing is to enhance its efficacy and/or reduce toxicity [6]. Additionally, processing is important to moderate reactions (such as anaphylaxis and allergies), diminish adverse events and modify detrimental properties (such as disagreeable flavors and odors) [6]. Experimental studies have been conducted to confirm the benefits of processing medicinal herbs to reduce their toxicity [7, 8].

RR is frequently processed using various methods to alter its efficacy or reduce side effects before clinical application. Some recommended methods involve RR steaming after dipping or soaking in alcohol, stir-frying RR with alcohol or vinegar or carbonizing [9]. Wang et al. compared the levels of anthraquinones and tannins in several processed RR extracts using different methods and found RR steaming decreased combined anthraquinones, while there was little change in total anthraquinones and tannins. Additionally, water-steamed RR decreased the anthraquinone glycoside content [7, 8]. Doui et al. reported that alcohol-soaking in 16 % ethanol and then steaming of RR decreased the sennoside content, while the processing increased the anthraquinone content [10, 11]. Most previous studies have focused on changes in the chemical constituents of processed RR.

Dongeuibogam, the most famous book on Korean traditional medicine, is an encyclopedic guideline of medical knowledge and treatment techniques first compiled by Joon Heo in 1613. As stated in this book, RR should be alcohol-steamed seven times when used for elderly patients [12]. In the present study, we evaluated the effects of seven-time alcohol-steaming on RR constituents and hepatotoxicity in rats and confirmed the statement in the book by providing experimental evidence. We measured the changes in constituents using reversed-phase high-performance liquid chromatography (RP-HPLC) and the effects on human hepatoma cell (HepG2) viability and body weight, blood chemistry and histopathological findings of rats subjected to various iterations of the RR alcohol-steaming process.

## Methods

### Materials

Dulbecco’s modified Eagle medium (DMEM), fetal bovine serum (FBS) and penicillin–streptomycin (P/S) were purchased from from Hyclone Laboratories, Inc. (Logan, UT, USA). Paraformaldehyde, 3-(4,5-dimethylthiazol-2-yl)-2,5-diphenyltetrazolium bromide (MTT), and hematoxylin were purchased from Sigma-Aldrich (St. Louis, MO, USA). Other reagents and solvents were of guaranteed or analytical grade.

### Preparation of RR extract

A dried rhizoma of *Rheum palmatum* was purchased from Kyung Hee Herb Pharm (Seoul, Korea); 500 g of unprocessed RR (RR-U) was soaked in 150 g of rice wine (14 %; Lotte Liquor BG, Seoul, Korea) for 2 h, steamed in a water bath for 2 h, then fully dried in an oven at 40 °C (RR-P1). The process was repeated four (RR-P4) or seven times (RR-P7). Each sample was deposited in the herbarium of the College of Pharmacy at Kyung Hee University (DGM P0, P1, P4 and P7). RR-U, RR-P1, RR-P4 and RR-P7 (RRs) were extracted using 70 % ethanol for 24 h at room temperature. After leaching, the mixture was passed through filter paper (Whatman No. 2; Korea), concentrated under reduced pressure using a rotary vacuum evaporator, and then freeze-dried (FDU-550R; Eyela Co., Japan) to powder. The yields were 24.80, 28.70, 26.70 and 27.61 %, respectively. RRs were kept at 4 °C. Before each experiment, the extracts were dissolved in an appropriate vehicle and were vortex-mixed for 2 min at room temperature.

### Analysis of RR constituents

The sennoside A, sennoside B and emodin constituents in RRs were determined using RP-HPLC. The HPLC system consisted of a HPLC unit (LC-20A HPLC instrument, Shimadzu Co., Japan) and reversed-phase Shiseido CAPCELL PAK C18 UG120S column (5 μm, 4.6 mm I.D. × 25 cm) was used for sennoside A detection and Biochoff chromatography column (ProntoSIL 250 × 4.6 mm) for sennoside B and emodin detection. For sennoside A detection, the mobile phase consisted of acetonitrile and 1.25 % acetic acid at a flow rate of 0.6 mL/min, with elution of 80 % acetic acid v/v at a flow rate of 1 mL/min at 30 °C. For sennoside B detection, the mobile phase consisted of acetonitrile and 0.05 M phosphoric acid at a flow rate of 1 mL/min, with gradient elution of 80 % phosphoric acid v/v at a flow rate of 1 mL/min at 40 °C. For emodin detection, the mobile phase consisted of methanol and 1 % phosphoric acid at a flow rate of 1 mL/min, with elution of 15 % phosphoric acid v/v at a flow rate of 1 mL/min at 40 °C. The injection volume was 10 μL.

### Effects of RRs on HepG2 cell viability

HepG2 cells were obtained from the Korea Cell Line Bank (KCLB, Seoul, Korea). HepG2 cells were maintained in DMEM supplemented with 10 % FBS, 1 % P/S in 95 air and 5 % CO_2_ at 37 °C. All experiments were performed 24 h after cells were seeded on 96-well plates at a density of 2.0 × 10^4^ cells/well. Cells were treated with 0.1–100 μg/mL RRs in serum-free media for 4 h. Cell viability was measured using the MTT method [13].

### Effects of RRs on rat liver

Thirty male Sprague–Dawley rats (5 weeks, 120–140 g) were obtained from Orient Bio (Sungnam, Korea). This study was carried out in accordance with the Principles of Laboratory Animal Care and Use Guidelines of Kyung Hee University, and was approved by the Ethics Committee of Kyung Hee University (Seoul, Korea). Animals were housed at an ambient temperature of 23 ± 1 °C and at a relative humidity of 60 ± 10 % under a 12 h light–dark cycle, and they were allowed free access to water and food. Animals were randomized into five groups: (1) control group; (2) RR-U treated group; (3) RR-P1 treated group; (4) RR-P4 treated group; (5) RR-P7 treated group. Vehicle or 3 g/kg/day of each sample dissolved in saline was administered orally once a day for 21 days. The treatment dose (3 g/kg) in rats was equivalent to the maximal clinical dose of RR in the Chinese pharmacopoeia (0.5 g/kg) [14, 15].

On the last day of treatment, blood samples were collected into non heparinized tubes and centrifuged at 3000 rpm for 10 min. The separated serum was analyzed to evaluate the liver enzymes. Alanine aminotransferase (ALT), aspartate aminotransferase (AST), alkaline phosphatase (ALP), total bilirubin (T-BIL), and gamma-glutamyltransferase (γ-GT) were entrusted to Chemon (Yongin, Korea) for analyses.

Necropsies were performed on all animals. After sacrifice, pieces of all excised tissues were individually placed in neutral buffered formalin for histologic examinations. Tissue specimens were processed into paraffin-embedded 5-μm sections and stained with hematoxylin and eosin (H&E). Sections were fixed in 100 % acetone at −20 °C for 15 min. Sections were stained with hematoxylin for 1 min and destained by dipping in acid ethanol. Then, sections were stained with eosin for 30 s, dehydrated and mounted. The images were photographed at 400× magnification using an optical light microscope (Olympus Microscope System BX51; Olympus, Tokyo, Japan).

### Statistical analysis

The statistical parameters were produced using GraphPad Prism 5.0 software. Values are presented as mean ± standard error of the mean (S.E.M.). Results were analyzed by one-way analysis of variance test with Tukey’s *post hoc* option.

## Results and discussion

In this study, we investigated the changes in the sennoside A, sennoside B and emodin constituents in RRs using RP-HPLC and found that seven-time alcohol-steaming reduced RR hepatotoxicity almost completely *in vitro *and *in vivo*.

**Fig. 1:**
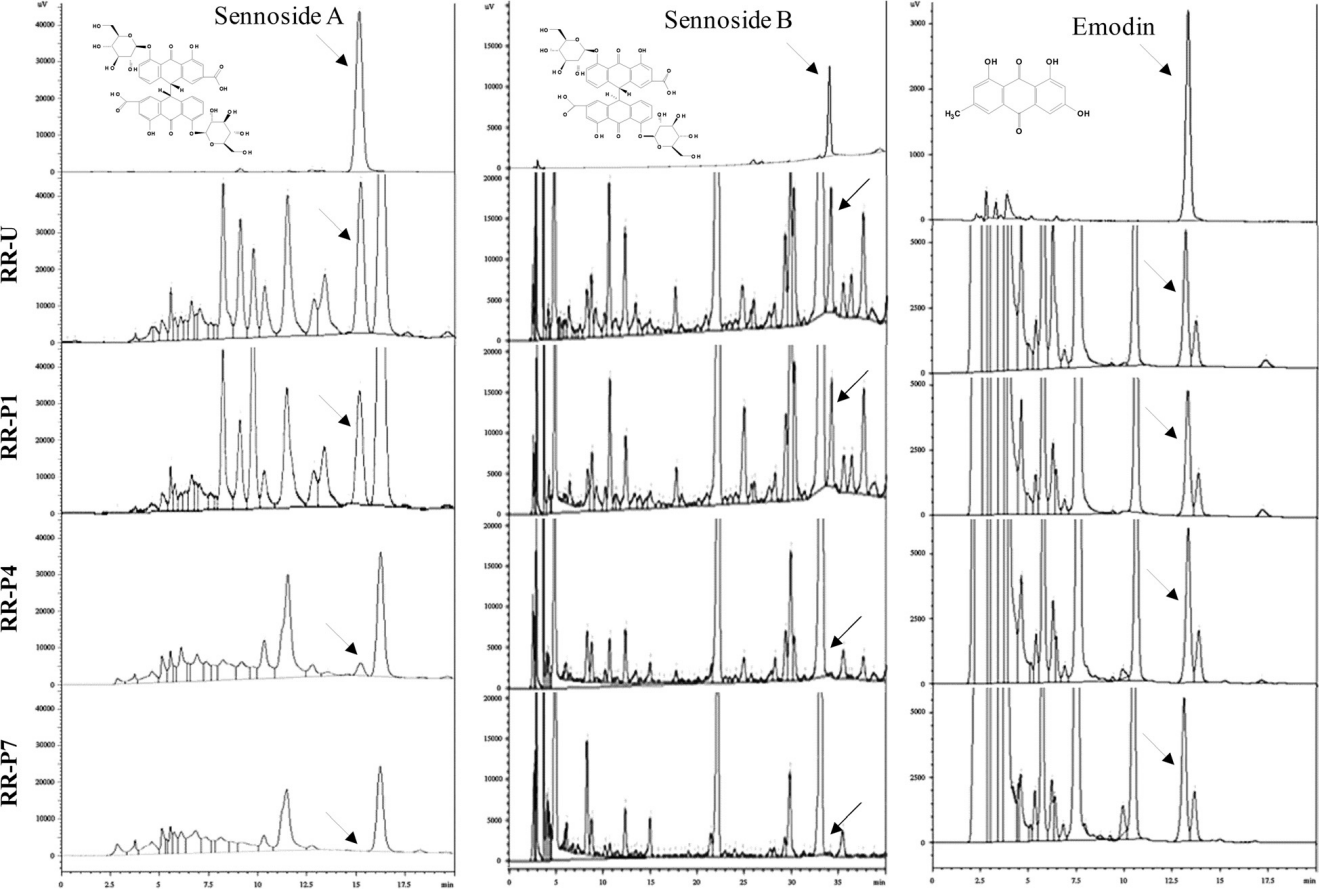
Sennoside A, sennoside B and emodin constituent changes in unprocessed or processed Rhei Rhizoma

Regarding changes in constituents after RR-U, RR-P1, RR-P4 and RR-P7, the levels of sennoside A were 13.7, 10.67, 1.62 and 0 μg/mg, respectively. The levels of sennoside B after RR-U, RR-P1, RR-P4 and RR-P7 were 8.9, 7.7, 4.6 and 3.8 μg/mg, respectively. The levels of emodin after RR-U, RR-P1, RR-P4 and RR-P7 were 5.9, 6.1, 6.3 and 5.8 μg/mg, respectively (Fig. 1). Doui et al. compared processing of RR with huangjiu or baijiu [10] and with or without preprocessing with alcohol [11] and speculated that steaming of RR decreased the sennoside contents; moreover, the reduction was greater as steaming time increased because steaming flexes the cells, causing elution of water-soluble compounds. In this study, alcohol-steaming of RR decreased the levels of sennoside A and B in an iteration-dependent manner, while emodin levels were unaffected. Noticeably, the sennoside peaks in RR-P7 almost disappeared.

**Fig. 2:**
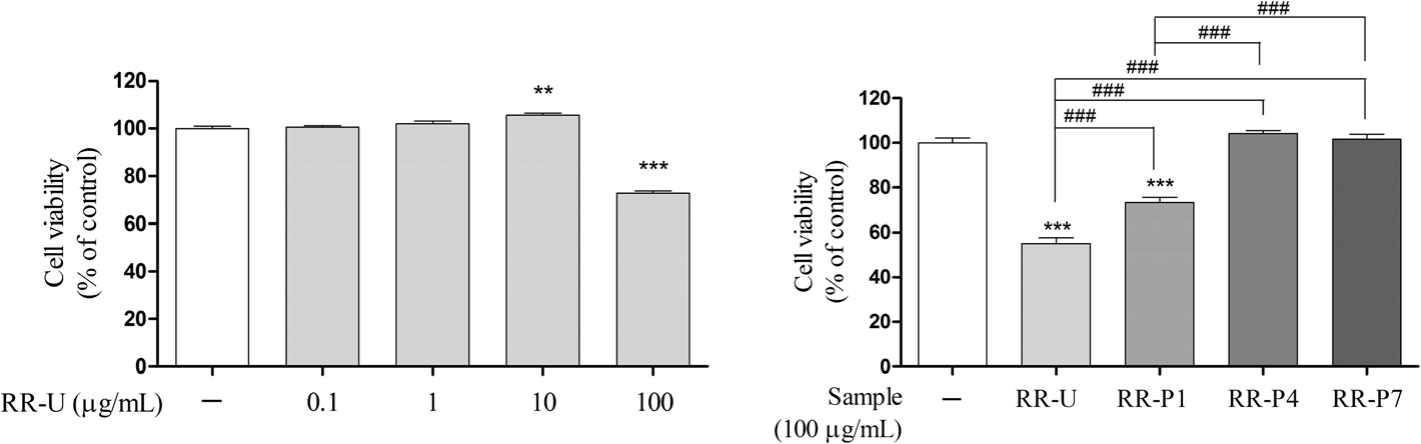
Effects of unprocessed or processed Rhei Rhizoma on HepG2 cell viability. Cells were treated with RR-U, RR-P1, RR-P4 and RR-P7 at concentration of 100 μg/mL for 4 h. Cell viability was presented as a percentage of the control. ^**^ *p* < 0.01 and ^***^ *p* < 0.001 compared with the control group. ^###^ *p* < 0.001 compared with RR-U or RR-P1 treated group

To evaluate the effect of alcohol-steaming on RR toxicity to HepG2 cells, MTT assay was performed. Significant cell toxicity was observed with 100 μg/mL treatment of RR-U. The toxicity of RR-U was recovered according to the number of alcohol-steaming process (Fig. 2).

**Fig. 3:**
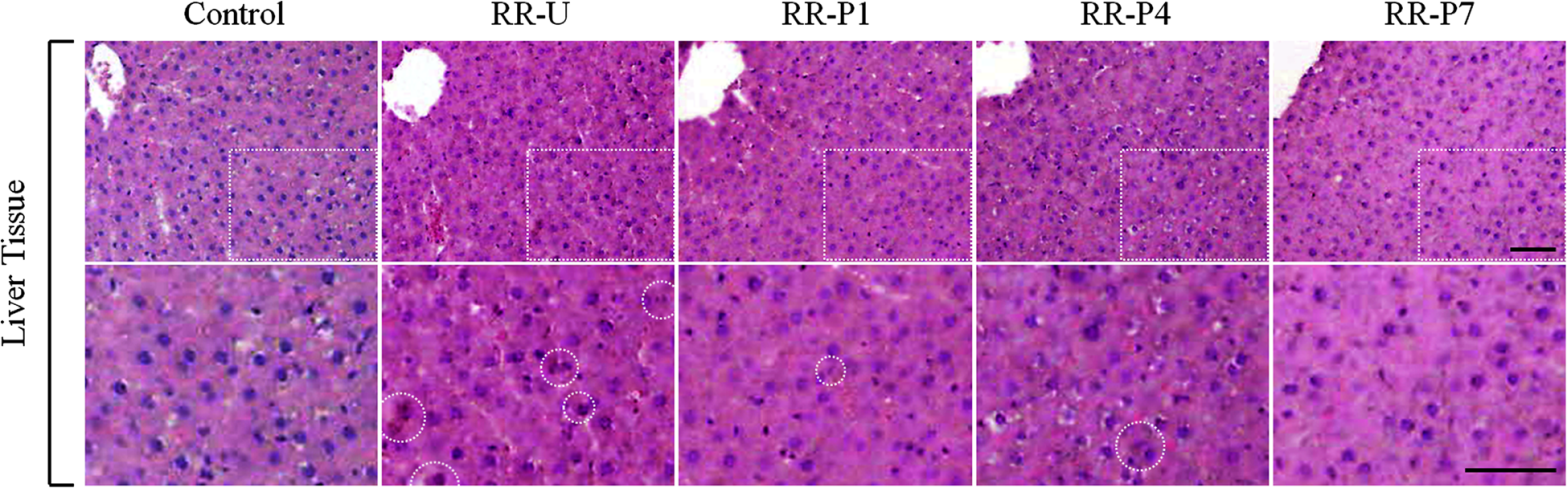
Liver morphologies of unprocessed or processed Rhei Rhizoma. Typical histopathological features of the rats treated with RR-U, RR-P1, RR-P4 and RR-P7. The yellowish brown bile pigments (white dashed circles). Scale bar = 50 μm

To confirm the effects *in vivo*, 3 g/kg of RRs were administered orally to rats for 21 days. The weights of body and liver in rats were measured to evaluate the general and liver-specific toxicity of RRs. The body weight in the RR-U group was significantly lower than that in the control group, while the body weight in the processed RRs groups increased significantly as the process was repeated. The relative weight (liver weight/body weight × 100) in the RR-U group was greater than that in the control group, and the weights in the RR-P1 and RR-P4 groups significantly decreased (Table 1). The levels of AST, ALT, ALP, T-BIL and γ-GT liver enzymes were measured to assess liver function. Serum liver enzymes are the most sensitive markers of hepatic damage because they are located in the cytoplasm and released into the circulation after cellular damage [16]. T-BIL and ALT levels in the RR-U group were higher than those in the control, RR-P4 and RR-P7 groups. The AST levels in the RR-U group were slightly higher, and those in the RR-P7 group were significantly lower, compared with the RR-U group. ALP and γ-GT levels differed among the groups, albeit not significantly so. Even though the reason for the result that RR-P7 group exhibited a slight increase in γ-GT compared to those of RR-P1 and RR-P4 groups was unclear, the increased level of RR-P7 group was statistically insignificant and still lower than that of RR-U group (Table 2). These findings also suggest that alcohol-steamed RRs affect the hepatobiliary and hematogenous systems. A histopathological study of rats treated with excessive RR-U showed swelling in the liver [4]. In this study, morphological changes in the liver were examined using H&E staining to evaluate the effect of RRs on liver toxicity. Stained liver tissues showed swelling and yellowish brown bile pigment indicating cholestasis in the rats treated with RR-U, while this phenomenon improved in the groups treated with processed RRs as the process was repeated (Fig. 3).

**Table 1:** Effect of unprocessed or processed Rhei Rhizoma on rat body and liver weights

	Control	RR-U	RR-P1	RR-P4	RR-P7
Body weight (g)	339.53 ± 6.54	290.48 ± 7.97^***^	326.83 ± 9.23^###^	333.77 ± 12.42^###^	339.18 ± 10.10^###^
Liver weight (g)	14.21 ± 0.58	14.17 ± 0.71	13.12 ± 1.07	13.03 ± 0.78	15.07 ± 0.92
Relative weight (%)	4.19 ± 0.25	4.88 ± 0.21^*^	4.01 ± 0.27^##^	3.90 ± 0.24^##^	4.44 ± 0.26

Relative weight: liver weight/body weight × 100. ^*^ *p* < 0.05 and ^***^ *p* < 0.001 compared with the control group

^##^ *p* < 0.01 and ^###^ *p* < 0.001 compared with RR-U treated group

**Table 2:** Effect of unprocessed or processed Rhei Rhizoma on biochemical indices of rats

	Control	RR-U	RR-P1	RR-P4	RR-P7
T-BIL (mg/dL)	0.05 ± 0.01	0.30 ± 0.07^***^	0.11 ± 0.01^##^	0.09 ± 0.01^###^	0.09 ± 0.01^##^
AST (IU/L)	94.15 ± 2.63	100.33 ± 6.44	86.52 ± 7.59	88.44 ± 6.72	81.92 ± 1.64^#^
ALT (IU/L)	44.38 ± 1.68	48.95 ± 2.01^*^	46.60 ± 4.37	42.62 ± 4.26^**###^	38.45 ± 1.28^*##^
ALP (IU/L)	301.18 ± 22.60	300.17 ± 31.66	257.45 ± 28.04	283.70 ± 25.88	293.73 ± 40.03
γ-GT (IU/L)	0.02 ± 0.02	0.14 ± 0.07	0.02 ± 0.02	0.02 ± 0.02	0.09 ± 0.05

^*^ *p* < 0.05, ^**^ *p* < 0.01, and ^***^ *p* < 0.001 compared with the control group. ^#^ *p* < 0.05, ^##^ *p* < 0.01, and ^###^ *p* < 0.001 compared with RR-U treated group

Furthermore, to investigate the efficacy of RRs after processing, we performed supplementary experiments to compare the anti-oxidative and neuroprotective activities of RRs. RRs exhibited strong radical scavenging activities, even anti-oxidative activities of RR-P7 were higher than those of the water extract of Scutellariae Radix (SBE), a positive control, which is well-known anti-oxidant and neuroprotectant [17, 18] (Additional file 1A). Also, we compared the protective effects of RRs against H_2_O_2_-induced neurotoxicity in PC12 cells. Decreased cell viability induced by H_2_O_2_ was inhibited by RRs pre-treatment, showing a better effect than SBE (Additional file 1B). These results suggest that the efficacy of RRs after processing may be still potent.

In this study, alcohol-steaming of RRs reduced their hepatotoxicity, which was normalized *in vitro* and *in vivo* after RR-P7 treatment due to the decreased the levels of sennoside A and B and maintained emodin levels. Sennosides have been reported to be related to hepatotoxicity [19]. Emodin, an anthraquinone derivative of RR, has anti-oxidant, anti-inflammatory and hepatoprotective effects [20–23]. Thus, the changes in chemical constituents suggest that the seven-time alcohol-steaming process reduced the hepatotoxicity of RR-U.

## Conclusions

The decreased hepatotoxicity of RR was related to the number of alcohol-steaming cycles; the seven-time alcohol-steaming process almost completely removed its hepatotoxicity. In this study, using modern pharmacological, toxicological and chemical analyses, we confirmed that RR required alcohol-steamed seven times and provided a scientific evidence for conducting systematic investigations of ancient documents.

## Additional file

Additional file 1:
**Radical scavenging activities and neuroprotective effects of unprocessed or processed Rhei Rhizoma.** (PDF 96 kb)
