# Supplementary material for: An experimental study on providing a scientific evidence for seven-time alcohol-steaming of Rhei Rhizoma when clinically used
Source: BMC Complement Altern Med. 2015 Oct 27;15:388. doi: 10.1186/s12906-015-0922-y (PMC4624659; doi:10.1186/s12906-015-0922-y)
Supplement: Additional file 1: — Radical scavenging activities and neuroprotective effects of unprocessed or processed Rhei Rhizoma. (PDF 96 kb) [file 12906_2015_922_MOESM1_ESM.pdf]

(A)

	**RR-U**	**RR-P1**	**RR-P4**	**RR-P7**	**SBE**
ABTS assay (IC_{50} value)	7.74 μg/mL	9.23 μg/mL	16.88 μg/mL	21.83 μg/mL	32.44 μg/mL
DPPH assay (IC_{50} value)	11.35 μg/mL	15.57 μg/mL	17.89 μg/mL	46.71 μg/mL	48.07 μg/mL

(B)

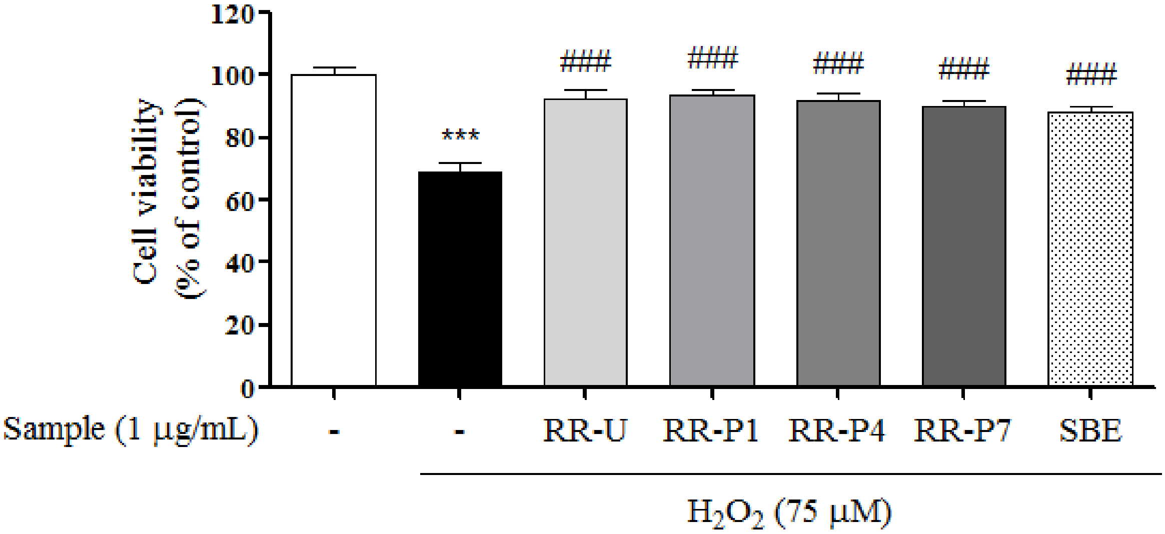

Supplementary Figure 1. (A) Radical scavenging activities of unprocessed or processed Rhei Rhizoma. (B) Neuroprotective effects of unprocessed or processed Rhei Rhizoma against H_2O_2 in PC12 cells. Cells were treated with RR-U, RR-P1, RR-P4, RR-P7 and SBE (1 μg/mL) for 1 h and incubated with H_2O_2 (75 μM) for a further 23 h. Cell viabilities are expressed as a percentage of the controls (cells treated with vehicle for 24 h). Values are indicated as the mean ± SEM. *** $p < 0.001$; mean values were significantly different from the control group. $^{\#\#\#}$ $p < 0.001$; mean values were significantly different from the H_2O_2 only treated group.
